# The Lightning Heart:  A Case Report and Brief Review of the Cardiovascular Complications of Lightning Injury

**Published:** 2010-09-05

**Authors:** William F McIntyre, Christopher S Simpson, Damian P Redfearn, Hoshiar Abdollah, Adrian Baranchuk

**Affiliations:** Cardiology Division, Kingston General Hospital, Queen's University, Kingston, Ontario, Canada

**Keywords:** Lightning Injury, Long QT, Myocardial Infarction, Takotsubo Cardiomyopathy

## Abstract

Lightning strike is a rare natural phenomenon, which carries a risk of dramatic medical complications to multiple organ systems and a high risk of fatality.  The known complications include but are not limited to: myocardial infarction, arrhythmia, cardiac contusion, stroke, cutaneous burns, respiratory disorders, neurological disorders, acute kidney injury and death.  We report a case of a healthy young man who suffered a lightning injury and discuss the cardiovascular complications of lightning injury, ranging from ECG changes to death.  The patient in our case, a 27-year old previously healthy male, developed a syndrome of rhabdomyolysis and symptomatic cardiogenic pulmonary edema. Electrocardiographic findings included transient T-wave inversions, late transition shift and long QT.  His clinical condition improved with supportive measures.

Early recognition of lightning injury syndromes and anticipation of complications may help us improve outcomes for these patients. Evaluation of patients having experienced a lightning injury should include a minimum of a detailed history and physical examination, 12-lead ECG and drawing of baseline troponins. Prolonged electrocardiographical monitoring (for monitoring of ventricular arrhythmias) and assessment for signs and symptoms of hemodynamic compromise may be warranted.

## Introduction

Lightning strike is a rare natural phenomenon which carries a risk of dramatic medical complications to multiple organ systems and a high risk of fatality [1].The known complications include but are not limited to: myocardial infarction, arrhythmia, cardiac contusion, stroke, cutaneous burns, respiratory disorders, neurological disorders, acute kidney injury and death.

We report a case of a young man struck by lightning and discuss the cardiovascular complications of lightning injury.

## Methods

Case report from our institution. Articles were selected from a computerized literature search in the Medline database using the key words: Lightning, Lightning Injuries, Electric Burns, Electric Injuries, Cardiac Arrhythmias, Contusions, Heart Injuries, Thoracic Injuries, Electrocardiography, Long QT, Myocardial Infarction, Myocardial Ischemia, Cardiology and all the possible combinations of the above. Two independent investigators (WFM, AB) reviewed the abstracts and selected the ones considered of interest for the review. Discrepancies were solved by consensus.

## Case Report

A 27 year-old male presented to hospital the day after a lightning strike complaining of nausea and vomiting, forearm pain, confusion, decreased urine output and shortness of breath.  He had no recollection of the injury. His past medical history was significant for infection with Hepatitis C virus, meningitis, cigarette smoking and previous intravenous drug use.  He had no pre-admission medications. His physical examination was unremarkable, save for 2nd-degree burns on both forearms.

Initial laboratory investigation revealed a creatine phosphokinase of 28 848 (55-197) U/L, creatinine 373 (0-106) mcmol/L, troponin I 1.103 (0.000-0.060) mcg/L and potassium 5.7 (3.5-5.2) mmol/L, consistent with a diagnosis of rhabdomyolysis.

**Figure 1:**
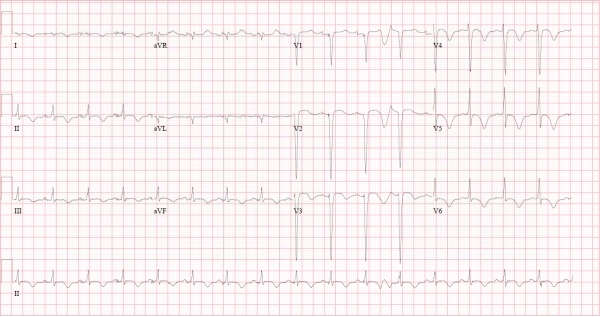

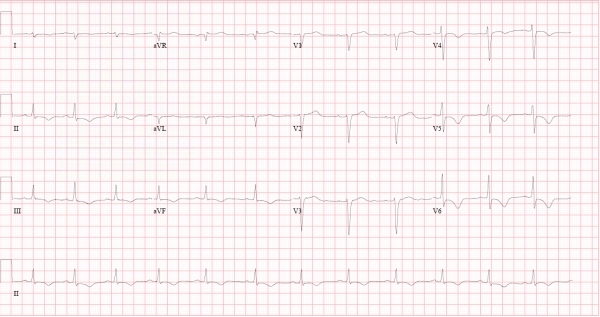
Twelve-lead electrocardiograms from a 27-year old male struck by lightning. Panel A (Above): ECG recorded approximately 24 hours after the event displaying diffuse T-wave inversion, late transition shift and long QT interval (QTc= 500 ms). Panel B (Below): Follow-up ECG 5 days later showing resolution of T-wave inversion in leads V1-V3, persistence of late transition shift  and a decrease in the QT interval to (QTc = 464 ms).

A 12-lead electrocardiogram (Figure 1) showed sinus rhythm at 98 bpm, T-wave inversion in the precordial and limb leads, late transition shift, rS pattern in leads I and aVL and long QT interval (QTc= 500 ms).

His transthoracic echocardiogram revealed moderate dilatation of the LV and mild systolic dysfunction (LVEF=53 %) with moderately elevated pulmonary artery pressures (58/33 mm Hg). There were no signs of a pericardial effusion.  Chest radiography demonstrated vascular cephalization without evidence of pulmonary edema.

The patient's acute kidney injury was treated with intravenous fluid and sodium bicarbonate administration. Urine output over the first 48 hours was 0.51 cc/kg/hr. On the 2nd post-admission day, the patient became acutely short of breath. Chest radiographs were consistent with pulmonary edema and the patient was treated symptomatically with intravenous furosemide.

A 12-lead ECG recorded on the 5th post admission day had had begun to normalize, displaying complete resolution of T-wave inversion in leads V1-V3, persistence of late transition shift and a decrease in the QT interval (QTc = 464 ms).The patient was discharged home on the 11th post-admission day with a creatine of 142 mcmol/L, creatinine phopshokinase of 0.131 U/L and potassium of 4.0 mmol/L and received no outpatient cardiac medications.

## Discussion

The preceding case details a previous relatively healthy 27-year old man who suffered a lightning injury.  He presented with confusion and cutaneous burns, and developed rhabdomyolysis and cardiogenic pulmonary edema.  Both of these insults resolved after administration of supportive measures. His 12-lead electrocardiogram demonstrated diffuse T-wave inversion, late transition shift and long QT interval, all of which were resolving or showing signs of resolution at the time of discharge.

Lightning is responsible for more fatalities each year on average than hurricanes and tornadoes combined. Only about 10% of lightning strike victims are killed; 90% survive. It is currently estimated that lightning causes 50 to 300 deaths per year in North America with four to five times as many victims suffering non-lethal injuries leading to severe and permanent disability [2]. Early recognition of lightning injury syndromes and anticipation of complications may help us improve outcomes for these patients. Among the most dramatic complications of lightning injury are those associated with the cardiovascular system. The cardiac complications associated with lightning injury are widespread range in severity from benign ECG changes to death  ( Table 1).

### Pathogenesis

Lightning injuries can occur from a direct strike, contact with a struck object or splash off of another object, as a result of ground currents or as blunt trauma from the lightning shockwave. Lightning is different from conventional electrical injuries in that it delivers a massive unidirectional impulse of current [2]. Multiple mechanisms have been proposed to explain the cardiovascular manifestations of lightning injury. These include the induction of coronary artery spasm, catecholamine-mediated effects, direct thermal damage, ischemia secondary to arrhythmia and coronary artery ischemia as part of a generalized vascular injury [3].

### Rhythm disturbances

Cardiac rate and rhythm can be affected both directly and indirectly as a result of lightning injury.  Electrical and mechanical trauma can have a direct effect, while autonomic stimulation and excessive catecholamine release may act indirectly.  The direct current of a lightning strike can cause cardiac depolarization and asystole [4]. Reports have been made of atrial arrhythmias, specifically atrial fibrillation as well as ventricular arrhythmias following lightning strike,  including occurring in previously healthy hearts.  As a general rule, these have tended to revert spontaneously to sinus rhythm over a period of days [5].

### Sudden Cardiac Death

A common cause of fatality in lightning injury is sudden cardiac death (SCD). Kondur et al. reported the case of a 75 year old man with an implanted cardioverter defibrillator (ICD) for primary prophylaxis of sudden death who was struck by lightning. His device-stored electrograms revealed ventricular fibrillation that was appropriately treated by a single ICD shock [7].  The sudden incidence of ventricular fibrillation may be most likely to occur with an indirect lightning strike to an object or the ground near the person, whereas a passage of direct current through the individual may be more likely to result in asystole [1].

### ECG Changes Including Long QT

A series of ECG changes have been shown as a result of lightning injury. These include specific and non-specific ST-segment changes, transient T-wave inversions, as well as lengthening of the QT interval and alterations in R-wave progression or late transition shift. Changes have also been seen that are consistent with ischemia, pericardial effusion, contusion and changes in repolarization.  Palmer reported a case of a previously healthy patient who developed transient prolongation of the QTc interval after being struck by lightning, becoming as long as 680 ms two days after admission and returning back to normal (QTc=410 s) over the course of a month [6].

Our patient developed a QTc of 500 ms, which normalized to 464 ms over the course of his admission. Such a change in repolarization could be a direct result of cellular injury.  [6] These cases suggest that QT interval prolongation could act as a substrate for previously mentioned ventricular arrhythmias. These findings highlight the importance for ECG monitoring in the seemingly-stable lightning-injured patient.

### Myocardial Contusion

Lightning strike can result in direct mechanical injury to the myocardium. This is often reflected biochemically with an increase in creatinine kinase and troponins.  As was the case with our patient, a number of patients after lightning strike have been shown to develop a clinical syndrome of hemodynamic deterioration and cardiogenic shock in the setting of systolic and diastolic dysfunction as demonstrated by echocardiography. Typically, these patients recover spontaneously after treatment with supportive measures, as did our patient.  These abnormalities are consistent with stunned myocardium or cardiac contusion.  The exact mechanism of abnormal contractility in the absence of direct electrofulguration is unknown but may be explained by release of oxygen free radicals, proteolysis of the contractile apparatus, and cytosolic overload of intracellular calcium, followed by reduced myofilament sensitivity to calcium [6].

In the case of our patient, he developed wall motion abnormalities with akinesis of some segments as demonstrated by echocardiography. Symptoms of heart failure were evident and there was a rise in biomarkers.  It is unclear in his case what contributions to his clinical condition may have come from myocardial ischemia, cardiac contusion and volume overload secondary to renal failure (due to rhabdomyolysis). However, the spontaneous recovery is suggestive of a transient mechanism, such as contusion.

### Myocardial Ischemia

Myocardial infarction and ischemia have been reported as a consequence of lightning injury in the absence of any thromboembolic coronary occlusion or vasoconstriction [9]. Our patient developed diffuse T-wave inversion and a rise in biochemical markers of myocardial injury after lightning strike and cardiogenic pulmonary edema that could represent a manifestation of ischemia. In this setting, the infarction could result from direct tissue damage from lightning strike (contusion) or lightning-induced vasospasm.

### Takotsubo Cardiomyopathy

Multiple case reports have emerged in the literature of development of Takostubo syndrome following lightning injury in previously healthy patients. This syndrome is characterized by transient apical and mid-left ventricle wall akinesis, may be accompanied with symptoms of heart failure and is usually self-resolving. Pathogenetic mechanisms that have been put forward to explain the development of this syndrome in the setting of lightning injury include a specific localized contusion or a consequence of vasospasm of the left anterior descending coronary artery [3]. In our patient, wall motion abnormalities were seen, but they were not compatible with Takostubo-type picture and they were better explained as a simple localized contusion.

## Conclusions

Lightning injury is a rare but potentially devastating condition. Patients who are struck by lightning can incur a range of cardiovascular complications.  Evaluation of these patients should include a minimum of a detailed history and physical examination, 12-lead ECG and drawing of baseline troponins. Prolonged electrocardiographical monitoring (for monitoring of ventricular arrhythmias) and assessment for signs and symptoms of hemodynamic compromise may be warranted.

## Figures and Tables

**Table 1:**
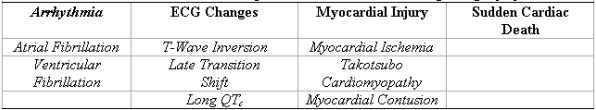
Cardiovascular Complications Associated With Lightning Injury
